# Does Intuition Cause Cooperation?

**DOI:** 10.1371/journal.pone.0096654

**Published:** 2014-05-06

**Authors:** Peter P. J. L. Verkoeijen, Samantha Bouwmeester

**Affiliations:** Department of Psychology, Erasmus University Rotterdam, Rotterdam, the Netherlands; University of Sao Paulo, Brazil

## Abstract

Recently, researchers claimed that people are intuitively inclined to cooperate with reflection causing them to behave selfishly. Empirical support for this claim came from experiments using a 4-player public goods game with a marginal return of 0.5 showing that people contributed more money to a common project when they had to decide quickly (i.e., a decision based on intuition) than when they were instructed to reflect and decide slowly. This intuitive-cooperation effect is of high scientific and practical importance because it argues against a central assumption of traditional economic and evolutionary models. The first experiment of present study was set up to examine the generality of the intuitive-cooperation effect and to further validate the experimental task producing the effect. In Experiment 1, we investigated Amazon Mechanical Turk (AMT) workers' contributions to a 4-player public goods game with a marginal return of 0.5 while we manipulated the knowledge about the other players' contribution to the public goods game (contribution known vs. contribution unknown), the identity of the other players (humans vs. computers randomly generating contributions) and the time constraint (time pressure/intuition vs. forced delay/reflection). However, the results of Experiment 1 failed to reveal an intuitive-cooperation effect. Furthermore, four subsequent direct replications attempts with AMT workers (Experiments 2a, 2b, 2c and Experiment 3, which was conducted with naïve/inexperienced participants) also failed to demonstrate intuitive-cooperation effects. Taken together, the results of the present study could not corroborate the idea that people are intuitively cooperative, hence suggesting that the theoretical relationship between intuition and cooperation should be further scrutinized.

## Introduction

Rand, Greene and Nowak [1] recently asked themselves the fundamental question “… whether people are predisposed towards selfishness, behaving cooperatively only through active self-control; or whether they are intuitively cooperative, with reflection and prospective reasoning favouring ‘rational’ self-interest.(pp. 428)”. In their paper, Rand and colleagues hypothesized that people develop strong cooperative intuitions because cooperation is typically rewarded in daily live. A crucial prediction of this social heuristics hypothesis is that people's intuitive reaction will be to behave cooperatively and that non-cooperative/selfish behavior emerges only after reflection. Consistent with the social heuristics hypothesis, Rand and colleagues found in a series of experimental and correlational studies that participants tend to behave more cooperatively under conditions promoting intuitive decision making than under conditions promoting reflective decision making. These findings are remarkable because they are clearly at variance with a historically influential philosophical position stating that people are self-centered acting socially only due to reflection/rational self-control. Additionally, the findings argue against a central assumption in traditional evolutionary models and economic models that people should display consistent behavioral styles (either cooperative or non-cooperative). Contrary to these models Rand and colleagues' findings suggest people can switch from one style to another dependent on their mind set (intuition vs. reflection). Hence, it might not come as a surprise that Rand and colleagues ‘paper has attracted quite a lot of attention from the scientific community but also from the popular press.

Crucial empirical support for Rand and colleagues’ [1] social heuristics hypothesis came from two experiments revealing a causal relationship between participants' monetary contribution to a common project in a one-shot public goods game. In Study 6, an internet experiment with Amazon Mechanical Turk participants, and in Study 7, an experiment in a psychological laboratory with college students, participants received a fee for taking part in the experiment and they were told they could earn a bonus as a result of the outcome of a one-shot public goods game. For the game, participants were given an additional amount of money and they had to decide how much of this money, if any, they wanted to contribute to a common project. Also, participants were informed they collaborated on this common project with three other unknown players of whom the contributions to the common project were not known. The bonus each of the four players received was calculated as follows: (additional money – own contribution) + 2*(sum of the contributions)/4. This implies that the highest personal payoff is obtained by defecting (i.e., contributing nothing to the common project) whatever the total contribution of the other three players is. After participants read the instructions on the one-shot public goods game they were taken to a decision screen. A random half of the participants were required to make a decision on their contribution within 10 seconds (time pressure condition: intuitive decision making), whereas the other half of the participants had to think and reflect at least 10 seconds before making their contribution (reflection/forced delay condition). In line with the social heuristics hypothesis, both experiments showed an *intuitive-cooperation effect*. That is, the mean contribution was significantly higher in the intuition/time-pressure condition than in the reflection/forced-delay condition.

## Experiment 1

Rand and colleagues [1] demonstrated the intuitive-cooperation effect through an experimental procedure in which participants were not aware of the team members' contributions. However, this situation is very uncommon in real-life cooperation. Therefore, the aim of Experiment 1 was to examine whether the intuitive-cooperation effect emerges under a more realistic experimental procedure in which participants are aware of the team members' contributions. On the one hand, one could argue that when the team members' contributions are known the social heuristics, i.e., the initial tendency to cooperate, are replaced by a different heuristic such as contributing “a fair share” to the common project. One the other hand, because social heuristics are assumed to have evolved due to extensive positive experience with collaboration, the intuitive-cooperation effect may be found for known and unknown team members' contributions.

In addition to examine the generality of the intuitive-cooperation effect, our first experiment aimed at further validating the social heuristics hypothesis. According to Rand and colleagues [1], social heuristics have developed as a result of cooperation with other humans. Therefore, it seems reasonable to assume that the social heuristics are triggered in economic games, such as the one Rand and colleagues [1] used, when participants collaborate with other people. However, when participants interact with computers that randomly generate contributions to a common project, it is unlikely they will use the social heuristics to make their contribution. Consequently, the social heuristics hypothesis predicts an intuitive-cooperation effect when participants are playing with humans but not when they are playing with computers. This prediction was tested in Experiment 1.

### Method

Below, we will present a summary of Experiment 1′s method and the results. For detailed information, we refer the reader to the Supporting Information (File S1).

In Experiment 1, participants played a one-shot public goods game identical to the one used by Rand and colleagues [1]. However, contrary to Rand and colleagues we explicitly stated the pay-off rule and we provided an example of a pay-off calculation. Furthermore, we created a 2×2×2 between-subjects design by manipulating the identity of *the team members* (humans vs. computers), *the contribution knowledge* (contribution of team members unknown vs. contribution of team members known), and *the decision constraint* (contribution under time pressure vs. contribution after a forced delay). We also assessed participants' *motivation* for their contribution and their quantitative *game understanding*.

### Results

For the statistical analyses reported in this paper, we used an alpha level of .05 as a threshold of statistical significance.

#### Contributions

Table 1 presents the relevant descriptive statistics of the contributions for participants who obeyed the time constraints. For the statistical test in Experiment 1, we submitted the contributions to a 2 *contribution knowledge* (contribution unknown vs. contribution known) × 2 *team members* (humans vs. computers) × 2 *decision constraint* (time pressure vs. forced delay) factorial analysis of variance (anova). The contributions of three participants from the *time-pressure* condition were excluded from the analysis, because these participants failed to meet the time constraint, i.e., 10 s, in this particular condition. Note that we excluded participants because we aimed at following Rand and colleagues' [1] analysis procedure. The sample means in the two human conditions revealed one intuitive-cooperation effect (contribution unknown) and one negative intuitive-cooperation effect (contribution known). Additionally, in both computer conditions we found that the mean contribution was higher in the *time-pressure* condition than in the *forced-delay* condition. However, the analysis failed to demonstrate significant effects (maximum *F*  =  3.28), and without any exception the effect sizes were extremely small (maximum partial eta squared = .012).

**Table 1:** Number of Participants (*n*), Mean (*M*), Standard Deviation (*Sd*) of Participants' Contributions (in dollar cents) and the 95% Confidence Interval (CI) as a Function of Team Member, Contribution Knowledge and Decision Constraint in Experiment 1.

						95% CI of the Mean	
Team member	Contribution knowledge	Decision constraint	*n*	*M*	*Sd*	Lower bound	Upper bound
Human	Unknown	Pressure	33	20.76	12.76	16.36	25.15
		Forced Delay	35	17.14	11.65	12.87	21.41
	Known	Pressure	40	21.00	13.83	17.01	24.99
		Forced Delay	39	22.10	13.00	18.06	26.15
Computer	Unknown	Pressure	30	24.07	12.30	19.46	28.68
		Forced Delay	43	22.26	13.52	18.41	26.11
	Known	Pressure	29	22.07	13.13	17.38	26.76
		Forced Delay	37	18.38	11.96	14.23	22.53

Note that the maximum contribution was 40 dollar cents.

#### Decision Times

Table 2 presents the relevant descriptive statistics of the decision times for participants who obeyed the time constraints. As expected, the mean and median decision times were lower in the *time-pressure* conditions than in the *forced-delay* conditions. It should be noted that our means and standard deviations were different from those reported by Rand and colleagues [1] in their Study 6 (i.e., Time pressure: *M* = 6.99, *Sd* = 2.06; Forced Delay: *M* = 34.83, *Sd* = 42.28, but judging from the latter large *Sd,* the mean decision time in the *forced-delay* condition might be due to participants who took a very long time to make their decision).

**Table 2:** Mean (*M*), Standard Deviation (*Sd*), Median (*Md*), Minimum (*Min*) and Maximum (*Max*) of the Decision Times in Experiment 1.

			Decision Time (in seconds)				
Team member	Contribution knowledge	Decision constraint	*M*	*Sd*	*Md*	*Min*	*Max*
Human	Unknown	Pressure	3.98	2.07	3.70	1.18	9.38
		Forced Delay	14.74	4.43	13.01	10.88	33.89
	Known	Pressure	3.80	1.97	3.05	1.06	10.32
		Forced Delay	15.54	8.86	12.42	11.03	60.56
Computer	Unknown	Pressure	3.58	1.47	3.33	1.63	7.95
		Forced Delay	13.76	3.11	12.53	10.99	24.66
	Known	Pressure	3.59	1.74	3.17	1.58	8.45
		Forced Delay	16.66	17.03	12.38	11.08	113.59

#### Quantitative understanding and motivation

After participants made their contribution, their quantitative understanding of the game's pay-off schedule was assessed. Quantitative understanding was measured by presenting participants with a hypothetical contribution scenario, i.e., four players each contributing 10 cents to the common project, along with the question to determine the bonus each participant would receive. The correct answer to the question was 50 cents. Only 10% of the participants provided this answer. A partially correct answer was 20 cents. In that case, a participant correctly added up the contribution of all four players, doubled the resultant contribution, and divided the outcome by 4. Yet, this participant did not add to these 20 cents the part of the initial amount of money that was left after the contribution, i.e., 30 cents. The 20-cents answer was given by 33% of the participants. Furthermore, 47% of the participants did not show game understanding. These participants answered the comprehension question with a bonus that was neither 50 cents nor 20 cents. The remaining 10% of the participants gave no answer or an unclear answer. Hence, if we apply a lenient criterion, a mere 43% of the participants (the percentages of the 50-cents category and 20-cents category combined) showed understanding of the game's pay-off schedule.

We should note that we performed the above presented 2 *contribution knowledge* (contribution unknown vs. contribution known) × 2 *team members* (humans vs. computers) × 2 *decision constraint* (time pressure vs. forced delay) factorial anova on the contributions of the 43% of the participants demonstrating game understanding. The outcomes of this anova as well as the conditions means were comparable to the results obtained with all participants.

We also asked participants to indicate the motivation underlying their contribution. Table 3 presents the motivation counts. The table contains the response category of 283 participants; 3 participants were not included because they did not give a motivation for their contribution. Two chi-square tests revealed a marginally significant relationship between motivation and decision constraint when humans were the team members, χ^2^(1) = 3.049, *p* = .089, odds ratio = 1.8, but not when computers were the team members, χ^2^(1) = 1.028, *p* = .311, odds ratio = 1.4. Contrary to the social heuristics hypothesis, the marginally significant chi-square test for humans indicated that 43% of the people in the *time-pressure* conditions reported cooperative motivations compared to 57% of the participants in the *forced-delay* conditions.

**Table 3:** Motivation Counts in as a Function of the Team Members and Decision Constraints in Experiment 1.

		Motivation		
		Cooperation	No Cooperation	Total (Row)
Human	Pressure	32	42	74
	Forced delay	41	30	71
Computer	Pressure	24	35	59
	Forced Delay	39	40	79
	Total (Column)	136	147	283

### Discussion Experiment 1

The goals of Experiment 1 were to examine whether the intuitive-cooperation effect generalizes to an experimental situation in which the team members' contributions are known, and whether the intuitive-cooperation effect disappears when participants interact with computers that randomly generate contributions. However, the results did not reveal any intuitive-cooperation effect: for each of the four *team member* x *contribution knowledge* combinations the difference between the mean contribution in the *time-pressure* (intuitive decision making) and the *forced-delay* condition (reflection) was small and non-significant. The failure to find an intuitive-cooperation effect in the unknown human condition was surprising because this condition was conceptually similar to Rand and colleagues' [1] Study 6, in which a clear intuitive-cooperation was found. Hence, in the unknown human condition, we did not replicate Rand and colleagues' intuitive-cooperation effect.

## Experiments 2a, 2b and 2c

The inconsistency between our results in the unknown human condition and the intuitive-cooperation effect in Rand and colleagues' [1] Study 6, may be due to unintended differences between their experimental procedure and materials and ours. One of these differences might be particularly relevant: we informed participants explicitly about the public goods game pay-off schedule and we included a pay-off calculation example in the instruction. However, it might be possible (see Rand and colleagues' Supplement Information for a similar argument) that these instruction features had induced reflective thinking in our participants, and this in turn might have erased the intuitive-cooperation effect.

Furthermore, in our experiment participants entered their contribution in a box, whereas participants in Rand and colleagues' [1] study used a slider. Also, in *the forced-delay* conditions of our Experiment 1, the timing of the contribution was experimenter controlled. By contrast, participants in Rand and colleagues' study had to keep track of the time themselves. Yet, we think it would be highly unlikely, and with respect to theoretical generalizability very undesirable when the intuitive-cooperation effect would turn out to depend on the way in which the contribution is made (typing a number in a box or using a slider) and/or the timing of the decision in the *forced-delay* condition (experimenter controlled or participant controlled).

In an attempt to replicate Rand and colleagues' [1] intuitive-cooperation effect, we conducted three experiments in which we sequentially changed the three abovementioned aspects of the procedure in our Experiment 1.These replications are important because they allow us to assess the reliability of the intuitive-cooperation effect with Mechanical Turk participants (see [2], [3], [4], [5], [6] and [7] for papers on replication in psychological research). If our instruction in Experiment 1 had indeed cancelled out the intuitive-cooperation effect due to the induction of a reflective decision mode, then the intuitive-cooperation effect should re-emerge in Experiments 2a, 2b and 2c.

### Method

For detailed information about the method in Experiments 2a, 2b and 2c, we refer the reader to the Supporting Information (File S1). In Experiment 2a, we copied the instruction and design from Rand and colleagues' [1] Study 6. However, contrary to Rand and colleagues' procedure, participants entered their contribution in a box, and decision timing in the *forced delay* condition was experimenter controlled. Experiment 2b was identical to Experiment 2a with the only exception that participants used a slider to make their contribution. Lastly, Experiment 2c was identical to Experiment 2b with the only exception that the timing of the contribution decision was participant controlled in the *forced-delay* condition.

### Results

#### Contributions and decision times

Table 4 presents the relevant descriptive statistics of the contributions in Experiments 2a, 2b and 2c for participants who obeyed the time constraints. Three independent *t*-tests were performed to test whether the mean contributions differed between the two conditions. None of these tests reached significance and the effect-sizes were small (all *p*'s>.453, all Cohen's *d*'s<.16).

**Table 4:** Number of Participants (*n*), Mean (M), Standard Deviation (*Sd*) of Participants' Contributions (in dollar cents) and the 95% Confidence Interval (CI) per Decision Constraint Condition for Experiments 2a, 2b and 2c.

					95% CI of the mean	
Experiment	Decision constraint	*n*	*M*	*Sd*	Lower Bound	Upper Bound
2a	Pressure	44	23.11	16.34	18.12	28.11
	Forced Delay	51	23.12	16.96	18.48	27.75
2b	Pressure	41	22.73	14.76	17.77	27.69
	Forced Delay	47	24.60	16.95	19.97	29.23
2c	Pressure	59	24.71	16.64	20.51	28.92
	Forced Delay	37	22.27	15.66	16.97	27.58

Note that the maximum contribution was 40 dollar cents.

In addition, Table 5 presents the relevant descriptive statistics of the decision times in Experiments 2a, 2b and 2c for participants who obeyed the time constraints. The mean and median decision times were lower in the *time-pressure* conditions than in the *forced-delay* conditions.

**Table 5:** Mean (*M*), Standard Deviation (*Sd*), Median (*Md*), Minimum and Maximum of the Decision Times per Decision Constraint Condition for Experiments 2a, 2b and 2c.

Experiment	Decision constraint	*M*	*Sd*	*Md*	Minimum	Maximum
2a	Pressure	3.49	1.43	3.27	1.17	7.40
	Forced Delay	15.09	5.97	13.11	11.18	39.73
2b	Pressure	3.76	2.06	3.00	1.08	9.76
	Forced Delay	15.28	6.47	12.62	10.95	37.98
2c	Pressure	5.82	2.31	5.61	2.16	10.16
	Forced Delay	27.55	27.43	20.59	9.52	175.76

### Discussion Experiments 2a, 2b and 2c

Like in Experiment 1, we failed to demonstrate an intuitive-cooperation effect in Experiments 2a through 2c. Hence, also in these experiments we did not replicate the intuitive-cooperation effect found by Rand and colleagues' [1] in their Study 6.

However, our replication failures are consistent with the findings from a recent paper by Rand, Peysakhovich, Kraft-Todd, Newman, Wurzbacher, Nowak and Greene [8]. Based on an overview of public good games studies they conducted with Mechanical Turk participants from the United States, Rand and colleagues showed that the intuitive-cooperation effect has declined and eventually disappeared since their first study (i.e., the study published in the 2012 *Nature* paper as Study 6). Furthermore, the outcomes of a survey distributed in April 2013 among United States Mechanical Turk workers demonstrated that the median number of self-reported participations in public good games was equal to 10. Consequently, it is reasonable to assume United States Mechanical Turk workers have experience with studies involving public good games.

This experience is relevant in the light of findings from Rand and colleagues' [1] Study 9. In that study, intuition was primed by asking participants to write a paragraph about a situation in which either their intuition had led them in the right direction, or careful reasoning had led them in the wrong direction. By contrast, reflection was primed by asking participants to write about either a situation in which intuition had led them in the wrong direction, or careful reasoning had led them in the right direction. Subsequently, participants had to decide on their contribution in a one-shot public goods game (i.e., the same game Rand and colleagues [1] used in their Study 6). After they made their contribution participants had to answer the following question: “To what extent have you participated in studies like this before? (i.e., studies were you choose how much to keep for yourself versus contributing to benefit others) “. Participants who chose the response “never” were classified as naïve; participants with other responses were classified as experienced. The results demonstrated that naïve participants' average contribution was higher when primed with intuition than when primed with reflection. Conversely, for experienced participants the mean contribution was similar in both conditions. To put it differently, the intuitive-cooperation effect was found for naïve participants but not for experienced participants. This interaction between experience and the intuitive-cooperation effect has been conceptually replicated by Rand and colleagues [8].

## Experiment 3

Considering that Mechanical Turk workers from the United States are experienced, and that the intuitive-cooperation effect interacts with experience, it might be possible that Rand and colleagues [8] recent failures to find an intuitive-cooperation effect in Mechanical Turk experiments as well as our failures to find such an effect in Experiment 1 and in Experiments 2a, 2b and 2c are due to the Mechanical Turk population becoming increasingly experienced in playing public good games. In order to test this experience hypothesis of our replication failures, we conducted a third experiment.

### Method

Experiment 3 was an exact replication of our Experiment 2c, but we only tested naïve participants. We refer the reader to the Supporting Information (File S1) for method details. If the experience explanation is correct and the intuitive-cooperation effect is only found for naïve participants, the intuitive-cooperation effect should show up in Experiment 3.

### Results

#### Contributions and decision times

A total of 109 Mechanical Turk workers took part in Experiment 3. Of these participants, 7 were excluded because they reported prior experience, 24 other participants were excluded because they did not meet the time constraints in their condition and 4 were excluded because they failed to enter a valid contribution. As a result, the following data analyses are based on the remaining 74 participants (38 in the *time-pressure* condition and 36 in the *forced-delay* condition). The mean contributions in the *time-pressure* condition was lower (*M* = 19.39, *Sd* = 15.72) than in the *forced-delay* condition (*M* = 21.73, *Sd* = 15.42). The results showed a reversed intuitive-cooperation effect and therefore a statistical analysis was omitted. Furthermore, the median decision times were respectively 5.17 in the *time-pressure* condition and 21.34 in the *forced-delay* condition.

### Discussion Experiment 3

The results from Experiment 3 were consistent with the results from the other experiments in this study. In Experiment 3, we again failed to demonstrate an intuitive-cooperation effect. This replication failure contradicts the experience hypothesis because it was obtained using naïve participants only.

One could argue that self-reports may not be the most valid measures of experience. This may be true, but by using a similar self-report as Rand and colleagues [1], [8] our findings can be compared to theirs. In addition, an anonymous reviewer proposed that Mechanical Turk workers might lie about their experience. However, we do not see why this would be the case. Perhaps, some participants ignored the admission criterion (i.e., naïve participants only) because they knew the participation fee was relatively high. Yet by using a post-experiment question we were able to filter out experienced participants. That is, at the end of the experimental session, we asked participants to indicate whether they had any experience with this kind of experiments AND we informed them they would receive their participation fee plus bonus independent of their answer. Considering there were no negative consequences associated with being honest, it seems unlikely that participants would lie in response to the post-experiment question. In fact, some participants were excluded from the experiment because they indicated after the experiment they were not naïve. In addition, a potential danger of the lying-participants argument is that it prevents the falsification of the theoretical framework relating experience to the intuitive-cooperation effect. Specifically, if self-reported naïve participants show an intuitive-cooperation effect they must have been honest about their experience, but if naïve participants fail to demonstrate intuitive-cooperation effect they must have been lying about their experience. According to Meehl [9] such post-hoc reasoning provides researchers with an easy escape from the *modus tollens* refutation and this in turn hampers scientific progress.

## Small Scale Meta-Analysis

**Figure 1:**
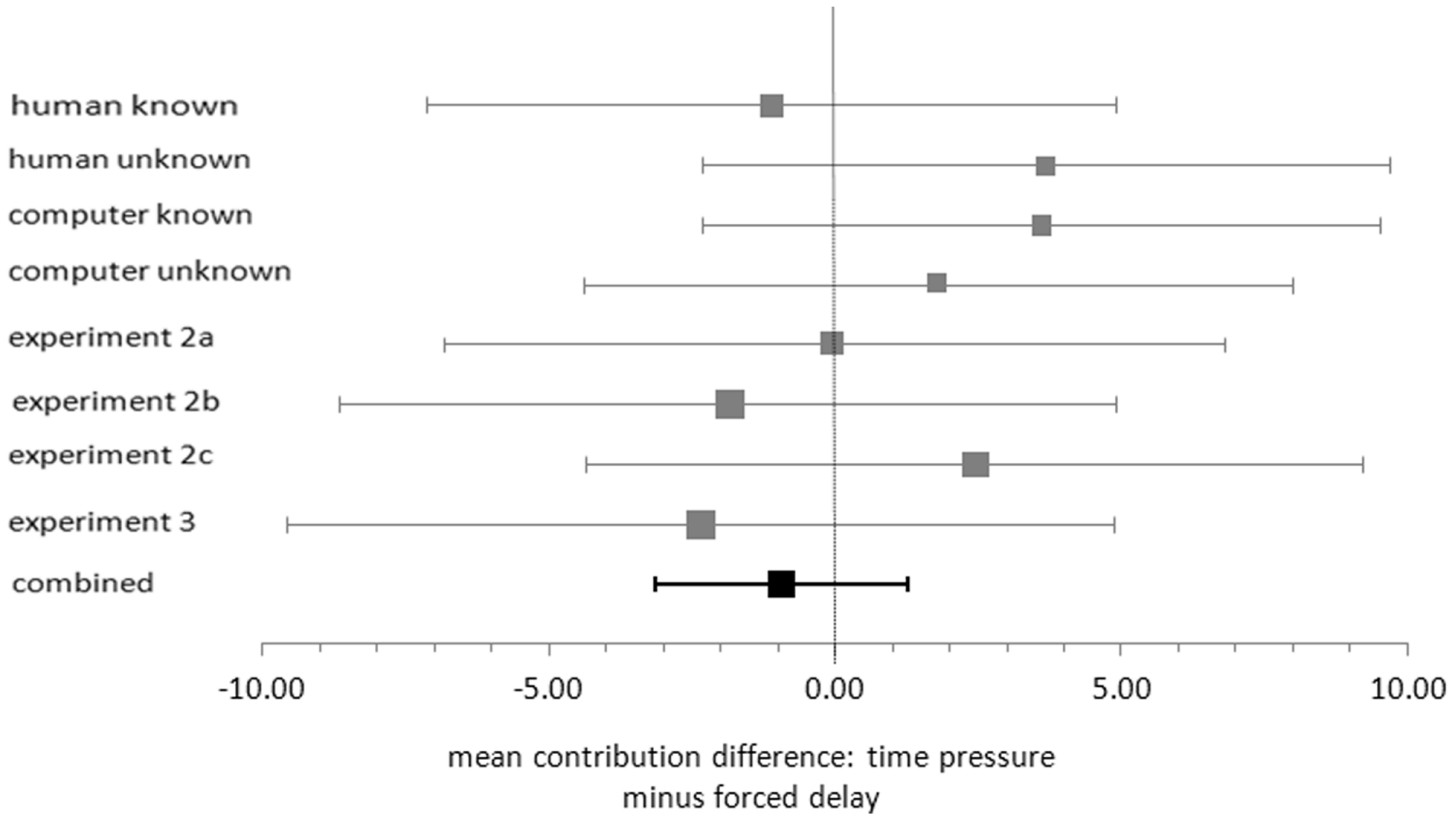
95% Confidence intervals of the mean difference between the *time-pressure* and the *forced-delay* condition in Experiment 1 (human known to computer unknown), Experiment 2a, 2b and 2c, and Experiment 3. The combined effect from the random-effects model is presented in the 95% Confidence interval at the bottom of the figure.

The present study resulted in eight estimates of intuitive-cooperation effect: four in Experiment 1, and one in Experiments 2a, 2b, 2c and 3. Inspired by Cumming's [10] “new statistics” approach we calculated a 95% confidence interval (CI) of the population mean for each of these estimates. Figure 1 presents the CIs in a forest plot. The squares in the forest plot represent the point estimate of the intuitive-cooperation effect parameter, i.e., the difference between the mean contribution in the *time-pressure* condition and the *forced-delay* condition (a positive difference denotes an intuitive-cooperation effect). One way to interpret CIs is that they indicate the precision of a parameter estimate. Given a particular scale of measurement, wide CIs reflect more uncertainty about the parameter than narrow CIs. The forest plot in Figure 1 demonstrates that the point estimates of the intuitive-cooperation effect vary, that each of the estimates is associated with a high degree of uncertainty (as evidenced by relatively wide CIs) and that the CIs show considerable overlap. The latter indicates there are no strong reasons to assume that the parameter estimates are based on samples from populations with different intuitive-cooperation effects.

The combined CI is based on a random-effects meta-analysis on the eight intuitive-cooperation effects from the present study. The combined CI is much narrower than the CIs of the separate experiments, and therefore it provides a more precise estimate of the intuitive-cooperation effect parameter. Furthermore, the combined point estimate shows a small negative intuitive-cooperation effect. Also, the combined CI includes the value of 0 indicating that the combined negative intuitive-cooperation effect is not statistically significant (at a two-tailed alpha level of .05).

## General Discussion

The present study contains three Mechanical Turk experiments in which we compared participants' contributions in a one-shot public goods game under *time-pressure* (intuitive decision making) and after a *forced-delay* (reflective decision making). The three experiments involved a total of eight comparisons, with four showing a positive intuitive-cooperation effect, three showing a negative intuitive-cooperation effect, and one showing a null effect. Additionally, the positive intuitive-cooperation effects were much smaller than the one reported by Rand and colleagues [1] in their Study 6. Furthermore, the combined effect that emerged from the meta-analysis revealed a small, non-significant negative intuitive cooperation effect. Hence, the outcomes of our experiments are inconsistent with Rand and colleagues' original finding.

Looking at the existing literature, the intuitive-cooperation effect appears to be rather variable. Rand and colleagues [1] found an intuitive-cooperation effect twice in respectively a Mechanical Turk experiment and in a psychological laboratory experiment. However, recently Tinghög, Andersson, Bonn, Böttiger, Josephson, Lundgren, Västfjäll, Kirchler, and Johannesson [11] failed to demonstrate intuitive-cooperation effects in three replications attempts (see their Experiment 5) of Rand and colleagues' Study 6. Furthermore, Rand and colleagues [8] showed that the intuitive-cooperation effect has declined over time and eventually disappeared in Mechanical Turk studies. Also, in the present study we failed to observe an intuitive-cooperation effect in three Mechanical Turk experiments.

Rand and colleagues [8] (see also Rand and Nowak [12]) propose that experience is an important moderator of the intuitive-cooperation effect. In addition, they suggest that the decline of the intuitive-cooperation effect in Mechanical Turk studies may be due to Mechanical Turk workers becoming increasingly experienced in public goods games. Empirical evidence in favor of the experience hypothesis comes from two studies (see Rand and colleagues [1], [8]) showing that the intuitive-cooperation effect occurs for naïve participants but not for experienced participants. However, at this point we are skeptical about the experience hypothesis because we have concerns about the validity of the experience measure and because we think the published data do not provide conclusive evidence for the experience hypothesis. Subsequently, we will elaborate on our concerns starting with the validity issue.

Rand and colleagues [1], [8] use self-reports to measure experience. Participants who indicate they never participated in public game studies before are considered naïve and participants who indicate they participated at least once in a public game study are considered experienced. We think there are a number of problematic aspects to this experience measure. For one, it does not appear to fit very well within the social heuristics hypothesis. According to this hypothesis, people develop strong cooperative intuitions because cooperation is typically rewarded in daily live. If this is true, then is it seems unlikely that only a single experience in laboratory situation is sufficient to distort a strong intuitive tendency. In addition, it seems reasonable to also take relevant experiences outside the lab into account when measuring experience. In fact, Rand and colleagues [1] show in Study 10 that experience in real life is indeed correlated with the intuitive cooperation effect. Furthermore, the experience of a participant with public good games experiments is the result of a range of factors, such as the number and variety of public good games in which the participant took part, the number and variety of other experiments, i.e., experiments that did not involve public good games, in which a participant took part apart (participating in these kind of experiments is likely to interfere with building up experience in public good games), what a participant learned from taking part in earlier public good games (this point is particularly relevant because in Rand and colleagues' study [1] as well as in our Experiment 1 a high percentage of participants actually failed to understand the task at hand), the time interval between successive participations (longer intervals are prone to result in participants forgetting the gist of the task), and the interval between the last participation and a current experiment. None of these factors are taken into account by Rand and colleagues' experience measure. Thus, all in all we think there are strong arguments to doubt the validity of Rand and colleagues' [1], [8] experience measure.

But even if we ignore the problems with the validity of the experience measure, the empirical evidence pertaining to the experience hypothesis is mixed. Rand and colleagues [1], [8] showed intuitive-cooperation effects with naïve participants. However, in the present study, we failed to find an intuitive-cooperation effect in Experiment 3, which was conducted with naïve Mechanical Turk participants. Similarly, Tinghög and colleagues [11] could not replicate an intuitive cooperation effect in three studies with samples of presumably naïve participants. The latter two findings are clearly inconsistent with the experience hypothesis.

## Conclusion

The experiments in the present consistently failed to demonstrate intuitive-cooperation effects. In addition, Experiment 3 showed that a failure to find an intuitive cooperation effect cannot be attributed to experience. Furthermore, given the problems with the validity of the experience measure used by Rand and colleagues [1], [8] and the mixed empirical support for the experience hypothesis, we think more research is required to shed light on the interaction between experience and the intuitive-cooperation effect.

## Supporting Information

File S1 **File S1 contains detailed descriptions of the method sections of Experiment 1, Experiments 2a through 2c and Experiment3.**
(DOCX)
