# Supplementary material for: Does Intuition Cause Cooperation?
Source: PLoS One. 2014 May 6;9(5):e96654. doi: 10.1371/journal.pone.0096654 (PMC4011763; doi:10.1371/journal.pone.0096654)
Supplement: File S1 — contains detailed descriptions of the method sections of Experiment 1, Experiments 2a through 2c and Experiment3. (DOCX) [file pone.0096654.s001.docx]

**Supporting Information**

**Ethics statement**

The following ethics statement applies to all experiments in the present study. In Dutch legislation the law on medical-scientific research on humans (Wet Medisch Wetenschappelijk Onderzoek met mensen; WMO) serves to protect people from medical maltreatment and experimentation. The WMO applies to research in which people are submitted to a medical/physical intervention or to research in which a certain mode of behavior is imposed on people (see, art. 1, lid 1, sub b of WMO). According to the WMO, approval from an ethics committee is not required for certain behavioral studies (note that it is almost always required for studies involving a medical/physical intervention).

Prior to running the present study, we consulted the chair of the Ethics Committee Psychology (ECP), Erasmus University Rotterdam, the Netherlands, to determine whether a formal approval of the committee was required. She concluded that a formal approval of the Ethics Committee was not necessary because the participants were given full-disclosure of the procedure (i.e., there was no deceit), participants received a payment proportionate to the task at hand, the experimental procedure was noninvasive and the results were analyzed anonymously. We did not ask the ECP for a formal written approval waiver, but we will obtain one if needed.

Furthermore, the participants in all experiments were United States citizens who were recruited online and voluntarily subscribed for participation in the described experiments. We did not obtain written consent.

**Experiment 1**

**Method**

*Participants.* 289 United States residents completed the experiment via the online work marketplace Amazon Mechanical Turk (MTurk; http://www.mturk.com). Recruiting participants via MTurk leads to a more diverse sample than the traditionally employed college undergraduate samples, and this in turn benefits the external validity of this experiment. The mean age of the 211 participants who choose to report their age was 31.58 years (*Sd =* 10.57, minimum 17, maximum 71). All participants reported their gender and their highest level of education. In the sample 39% of the participants were female and 61% were male. Furthermore, 11% of the participants had high-school or less as highest level of education, 75% of the participants indicated they either had some college, a 2-year college degree or a 4-year college degree, and the remaining 14% of the participants reported a Master’s degree, a Doctoral degree or a Professional degree (JD, MD) as highest level of education. Participants were randomly assigned to one of the conditions of a 2 *contribution knowledge* (contribution unknown vs. contribution known) x 2 *team members* (humans vs. computers) x 2 *decision constraint* (time pressure vs. forced delay) between-subjects design. Participants received $0.50 for taking part in the experiment. Each participant was only allowed to take part once. In addition, they could earn a bonus of up to $1. The height of the bonus was based on the outcome of the one-shot public goods game that we will describe subsequently.

*Procedure.* After accepting the hit in Amazon Mechanical Turk, participants were first asked to provide their age, gender and highest level of education. Subsequently, they entered a new screen in which they received the instruction for the one-shot public goods game. Rand and colleagues’ [1] instructions provided only implicit information about the pay-off schedule, and this might have resulted at least for some participants in their study in a limited understanding of the game. To make sure that participants had a good understanding of the game, we explicitly provided the pay-off schedule and we illustrated the pay-off schedule with a calculation. In Appendix A, we provide the literal instructions for the human-contribution-unknown, human-contribution-known, computer-contribution-unknown, and computer-contribution-known conditions. In the contribution-known conditions, participants were informed that the other players (humans or computers) had already contributed a total of 60 cents to the common project. By contrast, in the contribution-unknown condition, participants were not informed about the other team members’ contributions. In all conditions, the entire procedure took about 5 minutes.

Participants read the instructions at their own pace. After they finished reading the instructions, they clicked on a button to proceed to the contribution screen. For participants in the forced-delay conditions, a screen was shown with the text: *Please carefully consider you decision. You must wait and think for at least 10 seconds before making your decision.* This text was presented for 10 seconds after which the text disappeared and participants had to type in their contribution in a box. Thus, in the forced delay conditions, and contrary to the procedure in Rand and colleagues’ [1] study, timing of the decision was experimenter controlled. The rationale underlying this procedure choice was that we did not want to impose a dual task (i.e., keeping track of time) on the participants because such dual task might interfere with their reflective reasoning (the purpose of the forced-delay condition was to induce a reflective mode of processing). In the time-pressure condition, participants saw the following text on the contribution screen: *You have to decide within 10 seconds. Click on >> for your contribution*. When participants clicked on the >> button they could type in their contribution in a box. For all participants we recorded the time they took to make their contribution.

After participants made their contribution they were taken to the motivation screen. Subsequently, they were asked to briefly describe the motivation for their decision on how much to contribute. Subsequently, participants moved on the comprehension screen. This screen contained the following question: *Now, assume that you and each of the three other players/computers* (depending on the condition) *contributed 10 cents to the common project. Please indicate the magnitude of the bonus that you would receive in this situation.* Participants had to type in the answer (the correct answer is 50 cents) after which they were thanked for participating.

For the bonus payment, a participant was teamed up with 3 randomly selected participants from the pool of the remaining 288 participants. The participant then received the bonus according to the pay-off schedule described to them in the previous instructions.

*Scoring of motivation.* We also asked participants to indicate the motivation underlying their contribution. Each motivation was scored as either cooperative or non-cooperative. We defined a response as indicating a cooperative motivation if a participant indicated – in one way or the other – that he/she wanted to benefit the group as a whole. Examples of cooperative motivations are:

*“I wanted to contribute some in the hopes of benefiting my group, but also value self-preservation.”*

*“I wanted to give as much as possible so that the group would benefit.”*

*“I was hoping that other people would decide to put more in if we end up splitting at the end anyways.”*

By contrast we defined a response as non-cooperative if a participant reported – in one way or the other – selfishness as motivation or if a participant gave a response that was not related to cooperation or to selfishness. Examples of non-cooperative motivations are:

*“I will maximize my total earnings if I don’t contribute anything.”*

*“I want to make as much money as possible for myself.”*

*“I figure I'll get the $.50 for the study regardless, so I might as well gamble a little!”*

Two independent raters scored a subset of 40 random motivations. The inter-rater reliability was sufficient (Cohen’s Kappa = .67) for our purpose (i.e. comparing group averages).

**Experiments 2a, 2b and 2c**

**Method**

*Participants and design.* Table S1 shows the number of participants, the mean age, the gender and the highest education level of the people participating in Experiments 2a, 2b, and 2c. In all three experiments, participants played a one-shot public goods game. Within each experiment, participants were randomly assigned to either the *time-pressure* condition or the *forced-delay* condition. More information about the procedure and design is presented below. All participants were United States residents and they completed the experiment via the online work marketplace Amazon Mechanical Turk (MTurk; http://www.mturk.com). Participants received $0.50 for taking part in the experiment. In addition, they could earn a bonus of up to $1. Participants could only take part in one of the experiments, and they were not allowed to take part when they had already participated in Experiment 1.

*Materials and procedure.* In Experiment 2a, 2b and 2c we copied the design and procedure from Rand and colleagues [1]. Below, we present the exact instructions from Rand and colleagues’ Study 6 for the *time-pressure* condition and the *forced-delay* condition. These instructions (in italics) were taken from the Supplement Information provided with Rand and colleagues’ paper. The participants entered the experiment through the internet. On the first screen participants were told they would receive $0.50 for taking part in the experiment and that they could earn a bonus of up to $1 as the result of the outcome of the experiment. The literal information given to the participants was: *“Thank you for accepting this HIT. You have received $0.50 for participating. You also have the opportunity to receive additional money, which will be described in the next few pages.”*

Subsequently, on the second screen they received instructions for the one-shot public goods game they were about to take part in. The literal instructions were: *“You have been randomly assigned to interact with 3 other people. All of you receive this same* *set of instructions. You cannot participate in this study more than once. Each person in your group is given 40 cents for this interaction (in addition to the 50 cents you received already for participating). You each decide how much of your 40 cents to keep for yourself, and how much (if any) to contribute to the group’s common project (in increments of 2 units: 0, 2, 4, 6 etc.).*

*All money contributed to the common project is doubled, and then split evenly among the 4*

*group members. Thus, for every 2 cents contributed to the common project, each group member receives 1 cent. If everyone contributes all of their 40 cents, everyone’s money will double: each of you will earn 80 cents. But if everyone else contributes their 40 cents, while you keep your 40 cents, you will earn 100 cents, while the others will earn only 60 cents. That is because for every 2 cents you contribute, you get only 1 cent back. Thus you personally lose money on contributing.*

*The other people are REAL and will really make a decision – there is no deception in this study.*

*Once you and the other people have chosen how much to contribute, the interaction is over.*

*Neither you nor the other people receive any bonus other than what comes out of this interaction.”*

On the third screen participants were asked to make their contribution. The literal instruction was:

*“[Time pressure condition] Please make your decision as quickly as possible. You must make your decision in less than 10 seconds!*

*[Forced delay condition]. Please carefully consider your decision. You must wait and think for at least 10 seconds before making your decision.”*

In Experiment 2a, participants entered their contribution in a box (Rand and colleagues [1] used a slider in their Study 6), and decision timing in the *forced-delay* condition was experimenter controlled (this was participant controlled in Rand and colleagues’ Study 6). Experiment 2b was identical to Experiment 2a with the only exception that participants used a slider to make their contribution. Lastly, Experiment 2c used exactly the same procedure as Rand and colleagues did in their Study 6.

**Experiment 3**

**Method**

*Participants and design.* We recruited 108 United States Mechanical Turk participants who had never participated in studies like this before (see the next paragraph for more detailed information about the selection procedure). These participants were randomly assigned to either *time-pressure* condition or the *forced-delay* condition. More information about the procedure and design is presented below. Participants received $0.50 for taking part in the experiment. In addition, they could earn a bonus of up to $1.

*Materials and procedure.* In Experiment 3 we copied the procedure from Experiment 2c with two exceptions. First, prior to taking the experiment, we informed people in Mechanical Turk participant pool that this experiment involved a study in which a participant were to choose how much to keep for himself/herself versus contributing to benefit others. Subsequently, people were informed they were only allowed to take part in the experiment if they had never participated in studies like this before. It should be noted that this pre-experimental information was adopted from Rand and colleagues’ [1] Study 9, in which they compared the intuitive-cooperation effect for experienced and non-experienced participants. When people entered the experiment, the procedure was identical to the procedure used in Experiment 2c (and to the procedure used in Rand and colleagues’ Study 6 for that matter). After they had finished the experiment, participants were again asked to indicate whether they had ever participated in studies like this one before. In order to prevent participants from being dishonest, they were informed they would receive their participation fee plus bonus independent of their answer.

Table S1. Number of Participants (*n*), Mean Age (*M*) and standard deviation (*Sd*), Gender and Highest Education Level of the Participants in Experiments 2a, 2b, and 2c.

|  | Experiment | | |
| --- | --- | --- | --- |
|  | 2a | 2b | 2c |
| *n* | 124 | 91 | 127 |
| age *M*(*Sd*) | 31.91 (11.09) | 30.16 (8.88) | 32.21 (10.51) |
| % Female | 40 | 44 | 50 |
| % Education |  |  |  |
| High school or less | 18 | 17 | 15 |
| College | 74 | 76 | 76 |
| Degree | 6 | 7 | 9 |

**Appendix A**

Instructions in the experimental conditions of Experiment 1

*Instructions human-contribution-unknown*

Welcome to this little game! In this game you have been randomly assigned to interact with three other people. All of you receive this same set of instructions. You cannot participate in this study more than once. Each person in your group is given 40 cents for this interaction (in addition to the 50 cents you received already for participating). You each decide how much of your 40 cents to keep for yourself, and how much (if any) to contribute to the group’s common project. All money contributed to the common project is doubled, and then split evenly among the 4 group members.

Below we give an example of how the bonus is determined:

Suppose Pete contributes 10 cents to the common project, Andy contributes 20 cents, Mary contributes 20 cents, and John contributes 10 cents. This means that the total amount of money contributed to the common project is 10+20+20+10=60 cents. These 60 cents will be doubled, which results in a total of 120 cents for the common project. Now, these 120 cents will be equally divided among the four people. So everyone receives 30 cents from the common project. The bonus each person gets is these 30 cents PLUS the initial amount of money (40 cents) minus the individual contribution. For the four people in this example, this leads to the following bonus payments:

Pete: 30+(40-10)=60.

Andy: 30+(40-20)=50.

Mary: 30+(40-20)=50.

John: 30+(40-10)=60.

Remember that in this game you have been randomly assigned to three other people. The other people are REAL and will really make a decision – there is no deception in this study. You and the three other people are doing this task simultaneously; therefore you don't know what (if any) each of the people contributes.

*Instructions human-contribution-known*

Welcome to this little game! In this game you have been randomly assigned to interact with three other people. All of you receive this same set of instructions. You cannot participate in this study more than once. Each person in your group is given 40 cents for this interaction (in addition to the 50 cents you received already for participating). You each decide how much of your 40 cents to keep for yourself, and how much (if any) to contribute to the group’s common project. All money contributed to the common project is doubled, and then split evenly among the 4 group members.

Below we give an example of how the bonus is determined:

Suppose Pete contributes 10 cents to the common project, Andy contributes 20 cents, Mary contributes 20 cents, and John contributes 10 cents. This means that the total amount of money contributed to the common project is 10+20+20+10=60 cents. These 60 cents will be doubled, which results in a total of 120 cents for the common project. Now, these 120 cents will be equally divided among the four people. So everyone receives 30 cents from the common project. The bonus each person gets is these 30 cents PLUS the initial amount of money (40 cents) minus the individual contribution. For the four people in this example, this leads to the following bonus payments:

Pete: 30+(40-10)=60.

Andy: 30+(40-20)=50.

Mary: 30+(40-20)=50.

John: 30+(40-10)=60.

Remember that in this game you have been randomly assigned to three other people. The other people are REAL and will really make a decision – there is no deception in this study. Each of the three other people has already chosen their contribution. The total amount of money the three people contributed to the group’s common project is 60 cents. Your turn is next.

*Instructions computer-contribution-unknown*

Welcome to this little game! You have been randomly assigned to a game in which you can earn additional money. In this game you are playing with three computers located at the Erasmus University Rotterdam (the Netherlands). Each computer randomly contributes a certain amount of money between zero and 40 cents to a common project. You and each of the computers are given 40 cents for this interaction (in addition to the 50 cents you received already for participating). You each decide how much of your 40 cents to keep for yourself, and how much (if any) to contribute to the group’s common project. All money contributed to the common project is doubled, and then split in four. Below we give an example of how the bonus is determined:

Suppose computer A contributes 10 cents to the common project, computer B contributes 20 cents, computer C contributes 20 cents, and a participant contributes 10 cents. This means that the total amount of money contributed to the common project is 10+20+20+10=60 cents. These 60 cents will be doubled, which results in a total of 120 cents for the common project. Now, these 120 cents will be divided by four. So the participant receives 30 cents from the common project. The bonus the participant gets is these 30 cents PLUS the initial amount of money (40 cents) minus the participant’s individual contribution.

For the participant in this example, this leads to the following bonus payments:

Participant: 30+(40-10)=60.

Remember that in this game you have been randomly assigned to three computers. The three computers are REAL and will really make a contribution– there is no deception in this study. You and the three computers are doing this task simultaneously; therefore you don't know what (if any) each of the computers contributes.

*Instructions computer-contribution-known*

Welcome to this little game! You have been randomly assigned to a game in which you can earn additional money. In this game you are playing with three computers located at the Erasmus University Rotterdam (the Netherlands). Each computer randomly contributes a certain amount of money between zero and 40 cents to a common project. You and each of the computers are given 40 cents for this interaction (in addition to the 50 cents you received already for participating). You each decide how much of your 40 cents to keep for yourself, and how much (if any) to contribute to the group’s common project. All money contributed to the common project is doubled, and then split in four. Below we give an example of how the bonus is determined:

Suppose computer A contributes 10 cents to the common project, computer B contributes 20 cents, computer C contributes 20 cents, and a participant contributes 10 cents. This means that the total amount of money contributed to the common project is 10+20+20+10=60 cents. These 60 cents will be doubled which results in a total of 120 cents for the common project. Now, these 120 cents will be divided by four. So the participant receives 30 cents from the common project. The bonus the participant gets is these 30 cents PLUS the initial amount of money (40 cents) minus the participant’s individual contribution.

For the participant in this example, this leads to the following bonus payments:

Participant: 30+(40-10)=60.

Remember that in this game you have been randomly assigned to three computers. The three computers are REAL and will really make a contribution– there is no deception in this study. Each of the three computers has already chosen their contribution. The total amount of money the three computers contributed to the common project is 60 cents. Your turn is next.
